# Interferon-gamma in the first-line therapy of ovarian cancer: a randomized phase III trial

**DOI:** 10.1054/bjoc.1999.1053

**Published:** 2000-02-21

**Authors:** G H Windbichler, H Hausmaninger, W Stummvoll, A H Graf, C Kainz, J Lahodny, U Denison, E Müller-Holzner, C Marth

**Affiliations:** Department of Obstetrics and Gynaecology, University Hospital, Anichstrasse 35, Innsbruck, A-6020, Austria; Departments of Medical Oncology, Obstetrics and Gynaecology, General Hospital, Muellner-Hauptstrasse 48, Salzburg, A-5020, Austria; Department of Gynaecology, Hospital Barmherzige Schwestern, Seilerstaette 4, Linz, A-4020, Austria; Department of Gynaecology, University Hospital, Waehringer Guertel 18–20, Vienna, A-1090, Austria; Department of Gynaecology, General Hospital, Probst-Führerstrasse 4, St Poelten, A-3100, Austria; Department of Obstetrics and Gynaecology, Wilhelminenspital, Pavillon 28, Montleartstrasse 37, Vienna, A-1160, Austria

**Keywords:** randomized phase III trial, multicentre study, ovarian cancer, interferon-type II

## Abstract

Intraperitoneal treatment with interferon-γ (IFN-γ) has been shown to achieve surgically documented responses in the second-line therapy of ovarian cancer. To assess its efficacy in the first-line therapy, we conducted a randomized controlled trial with 148 patients who had undergone primary surgery for FIGO stage Ic–IIIc ovarian cancer. In the control arm women received 100 mg m^−2^cisplatin and 600 mg m^−2^cyclophosphamide, the experimental arm included the above regimen with IFN-γ 0.1 mg subcutaneously on days 1, 3, 5, 15, 17 and 19 of each 28-day cycle. Progression-free survival at 3 years was improved from 38% in controls to 51% in the treatment group corresponding to median times to progression of 17 and 48 months (*P* = 0.031, relative risk of progression 0.48, confidence interval 0.28–0.82). Three-year overall survival was 58% and 74% accordingly (n.s., median not yet reached). Complete clinical responses were observed in 68% with IFN-γ versus 56% in controls (n.s.). Toxicity was comparable in both groups except for a mild flu-like syndrome, experienced by most patients after administration of IFN-γ. Thus, with acceptable toxicity, the inclusion of IFN-γ in the first-line chemotherapy of ovarian cancer yielded a benefit in prolonging progression-free survival. © 2000 Cancer Research Campaign


*British Journal of Cancer* (2000) **82**(6), 1138–1144
© 2000 Cancer Research Campaign
Article no. bjoc.1999.1053, available online at http://www.idealibrary.com on IDEAL®

### Interferon-gamma in the first-line therapy of ovarian cancer: a randomized phase III trial

**GH Windbichler[1], H Hausmaninger[2], W Stummvoll[3], AH Graf[4], C Kainz[5], J Lahodny[6], U Denison[7], E Müller-Holzner[1] and C Marth[1]**

[1]Department of Obstetrics and Gynaecology, University Hospital, Anichstrasse 35, A-6020 Innsbruck, Austria; Departments of [2]Medical Oncology and [4]Obstetrics and Gynaecology, General Hospital, Muellner-Hauptstrasse 48, A-5020 Salzburg, Austria; [3]Department of Gynaecology, Hospital Barmherzige Schwestern, Seilerstaette 4, A-4020, Linz, Austria; [5]Department of Gynaecology, University Hospital, Waehringer Guertel 18–20, A-1090 Vienna, Austria; [6]Department of Gynaecology, General Hospital, Probst-Führerstrasse 4, A-3100 St Poelten, Austria; [7]Department of Obstetrics and Gynaecology, Wilhelminenspital, Pavillon 28, Montleartstrasse 37, A-1160 Vienna, Austria

**Summary** Intraperitoneal treatment with interferon-γ (IFN-γ) has been shown to achieve surgically documented responses in the second-line therapy of ovarian cancer. To assess its efficacy in the first-line therapy, we conducted a randomized controlled trial with 148 patients who had undergone primary surgery for FIGO stage Ic–IIIc ovarian cancer. In the control arm women received 100 mg $m^{-2}$ cisplatin and 600 mg $m^{-2}$ cyclophosphamide, the experimental arm included the above regimen with IFN-γ 0.1 mg subcutaneously on days 1, 3, 5, 15, 17 and 19 of each 28-day cycle. Progression-free survival at 3 years was improved from 38% in controls to 51% in the treatment group corresponding to median times to progression of 17 and 48 months ($P = 0.031$, relative risk of progression 0.48, confidence interval 0.28–0.82). Three-year overall survival was 58% and 74% accordingly (n.s., median not yet reached). Complete clinical responses were observed in 68% with IFN-γ versus 56% in controls (n.s.). Toxicity was comparable in both groups except for a mild flu-like syndrome, experienced by most patients after administration of IFN-γ. Thus, with acceptable toxicity, the inclusion of IFN-γ in the first-line chemotherapy of ovarian cancer yielded a benefit in prolonging progression-free survival. © 2000 Cancer Research Campaign

**Keywords:** randomized phase III trial; multicentre study; ovarian cancer; interferon-type II

Ovarian cancer is one of the major causes of cancer death in women and the leading cause among gynaecological malignancies. The concurrent standard of treatment for these patients after initial surgery consists of a platinum-based chemotherapy. Recently, McGuire et al (1996) could demonstrate that inclusion of paclitaxel into the first-line therapy resulted in improved overall survival (OS). Although such advances in treatment have improved outcome, survival of patients diagnosed with advanced disease is still poor (Ozols et al, 1997). In the effort to find new therapeutic modalities, we focused our interest on biologic modifiers such as interferons (IFNs) because of their well defined pleiotropic biological actions (Billiau, 1996). IFN-γ, which is produced by activated T-cells and natural killer (NK) cells exerts antiproliferative effects on neoplastic cells including ovarian cancer cells, as demonstrated by Saito et al (1986). It is essential for tumour rejection in animal models (Malik et al, 1991; Dighe et al, 1994) and leads to enhanced immunogenicity of tumour cells, probably mainly by induction of cell surface antigens (Kaplan et al, 1998).

Particularly, all types of IFNs augment MHC class I expression, which has also been demonstrated for ovarian cancer cells, and IFN-γ, but not type I IFNs, induces a marked increase in MHC class II antigens (Marth et al, 1996) and in adhesion molecules. IFN-γ is further known to up-regulate surface expression of CA 125 (Marth 1989), which also could modulate targetability for corresponding antibodies or sensitized T-cells. Another mechanism of inhibition of tumour cell proliferation might be the ability of IFN-γ to down-regulate the message and encoded protein of the proto-oncogene HER-2/neu as demonstrated for ovarian cancer cells by Marth et al (1990). Increased expression of this membrane protein with structural homology to the epidermal growth factor receptor possibly provides a selective advantage in proliferation and has been found to be associated with poor prognosis and cisplatin resistance (Hancock et al, 1991). In a previous study we found that IFN-γ decreased mRNA expression of HER-2/neu, even in cisplatin-resistant cells, although it did not restore cisplatin sensitivity (Marth et al, 1997).

Moreover, modulatory effects of IFN-γ on cells of the immune system include the stimulation of NK cells and macrophages. The augmentation of cytotoxicity of macrophages was demonstrated by Mannel and Falk (1983) under preclinical, and Allavena et al (1990) under clinical, conditions.

Interleukin (IL)-12, the essential mediator of $T_H1$ differentiation in naive T-cells directly induces IFN-γ gene transcription, which is itself a major characteristic and effector molecule of the $T_H1$ cytokine response. IFN-γ, in turn, helps maintain a $T_H1$ cytokine profile and the associated cellular immune response by reciprocal induction of IL-12, thus forming a positive feedback loop (Boehm et al, 1997). Although these properties make IFN-γ an attractive potential anticancer agent, clinical studies for ovarian cancer have been limited to only a few trials. Mostly, an intraperitoneal (i.p.) route was chosen, because the disease mostly remains confined to

*Received 24 May 1999*
*Revised 13 September 1999*
*Accepted 24 September 1999*

*Correspondence to:* C Marth the peritoneal cavity even in advanced stages or during disease progression and locally higher concentrations of the agent are achievable by i.p. administration. While earlier trials with i.p. IFN-γ and limited numbers of patients yielded contradictory results (D'Acquisto et al, 1988; Allavena et al, 1990; Colombo et al, 1992), recently Pujade-Lauraine et al (1996) reported an overall response rate of 31% in a series of 108 patients with residual disease at second-look laparotomy after cisplatin-based first-line chemotherapy. Intraperitoneal IFN-γ was administered twice weekly in a dose of $20 \times 10^6$ IU $m^{-2}$. Of 71 patients who were re-evaluated surgically, women who were less than 60 years old and had a residual tumour of less than 2 cm at initiation of IFN treatment ($n = 42$) achieved an objective response rate of 54%.

Only a few trials for ovarian cancer have been conducted using systemically applied IFN-γ. Welander et al (1988) reported a high-dose regimen (2 mg $m^{-2}$ daily intravenously (i.v.)) to be active in relapsing ovarian cancer with 4/14 patients responding. However, preclinical studies found a dose of 0.1 mg effective in stimulating immunological responses. Maluish et al (1988) demonstrated monocyte and NK cell activation by subcutaneous (s.c.) or intramuscular (i.m.) application at this level. Kleinerman et al (1986) tested the stimulation of monocyte-mediated cytotoxicity from patients undergoing phase I trials with IFN-γ. Treatment with a dose of 1 mg $m^{-2}$ i.m. did not produce anti-tumour activity, while in the same patients cytotoxic monocyte function was restored when the dose of IFN-γ was reduced to 0.5 mg $m^{-2}$. Aulitzky et al (1987) used neopterin and β2-microglobulin as markers to monitor the biological response to IFN-γ in patients with renal cell carcinoma. IFN-γ in a dose range of 0.01–0.5 mg produced strong dose-dependent up-regulations of both markers after the first injection. However, the neopterin response was down-regulated with repeated injections at all dose levels. Markedly reduced β2-microglobulin levels were seen with repeated application of 0.5 mg IFN-γ, the highest dose tested. Thus, although monocyte–macrophage activation is well documented, higher doses and repeated continuous applications may lead to immunosuppression possibly by counter-regulatory effects.

In vitro data further demonstrated synergistic inhibition of proliferation of ovarian cancer cells in treated simultaneously with cisplatin and IFN-γ (Nehmé et al, 1994).

With this background we initiated a randomized phase III trial to evaluate the therapeutic potential of IFN-γ in the first-line therapy of ovarian cancer given together with a cisplatin-based chemotherapy. A low-dose IFN-γ scheme with 0.1 mg s.c. three times a week on alternate weeks was chosen to circumvent a possible immunosuppression by continuous application and high doses. For ovarian cancer this is the first trial with IFN-γ in the first-line therapy and concomitantly to chemotherapy.

### PATIENTS AND METHODS

### Patients

This prospective randomized study was performed in 12 Austrian gynaecological and medical centres between December 1991 and March 1998 with enrolment of patients until March 1997. The study protocol was approved by the Ethical Committee of the University of Innsbruck Medical School as well as the respective committees of the other participating centres. All patients gave their written informed consent before enrolment.

Eligible patients had undergone primary surgical debulking of histologically confirmed invasive epithelial ovarian cancer in FIGO stages Ic–III. Patients in FIGO stage IV could be included if this was due to malignant pleural effusion only. Additional eligibility criteria were WHO performance status 0–2, adequate renal function (creatinine clearance ≥ 60 ml $min^{-1}$), sufficient hepatic function (bilirubin ≤ 1.5 mg $dl^{-1}$), WBC count ≥ 3 g $l^{-1}$, platelet count ≥ 100 g $l^{-1}$, and age less than 75 years. Moreover, patients with concomitant severe cardiovascular disease, life expectancy of less than 3 months, patients with a recent second malignancy or history of thrombo-embolic disease were excluded. Patients were enrolled up to 4 weeks after initial surgery, but before any other treatment.

### Randomization and treatment

Randomization was stratified by treatment centre and the amount of residual tumour after surgery (< 2 cm vs ≥ 2 cm). The random-allocation lists for each treatment centre were computer-generated at entry into the study. Patients were allocated to a treatment arm by the study centre by means of fax transmission.

To avoid interobserver variability in tumour grading, all available slides were reviewed by one pathologist (EM-H).

Eligible patients were randomly assigned to receive either a standard chemotherapy regimen consisting of cisplatin and cyclophosphamide (CP), given every 4 weeks or the same chemotherapy regimen on the second of 28 days combined with recombinant IFN-γ 0.1 mg in a fixed dose administered s.c. on days 1, 3, 5 and 15, 17, 19.

Patients in both treatment groups received i.v. cisplatin 100 mg $m^{-2}$ body-surface area parallel to cyclophosphamide 600 mg $m^{-2}$. Courses of CP or CP plus IFN-γ were repeated every 4 weeks for a total of six cycles unless disease progression or limiting toxicity occurred or patients refused further treatment. In case of myelodepression with white cell counts of less than 3 g $l^{-1}$ or platelet counts of less than 100 g $l^{-1}$, therapy could be delayed for a maximum of 3 weeks. If myelopoiesis did not regenerate during this time, treatment was continued with cisplatin only. In the case of a nadir of leucocytes < 1 g $l^{-1}$ or platelets < 20 g $l^{-1}$ the cyclophosphamide dose was reduced by 25%.

### IFN treatment

Human recombinant IFN-γ (a donation of Bender, Vienna, Austria) was administered s.c. in a fixed dose of 0.1 mg three times weekly every other week (corresponding to $2 \times 10^6$ IU).

According to protocol interferon treatment was discontinued in the event of grade 4 toxicities other than alopecia or myelosuppression and in the case of newly diagnosed thrombo-embolic disease.

Premedication with paracetamol 1 g was allowed to ameliorate IFN-induced fever or other flu-like symptoms.

### Clinical assessments and response criteria

At baseline and after six courses of therapy a physical examination was performed together with a blood count, serum CA 125 and blood chemical measurements, chest radiography and computerized tomography (CT) scan. In addition, blood counts and chemical measurements were performed at the start of each cycle.

© 2000 Cancer Research Campaign 

Response was assessed by clinical evaluation. A second-look operation after completion of therapy was not obligatory, and if performed by the attending clinician, the result was not taken into account for evaluation of response. Patients with residual disease or tumour progression during therapy were considered evaluable for response. A complete clinical response (CR) was defined as no clinical evidence of disease after completion of therapy according to the results of the gynaecological examination, CT scan and CA 125 serum levels. A partial response (PR) was taken to be tumour regression ≥ 50% and progressive disease (PD) tumour growth ≥ 25%. No change (NC) was defined as regression < 50% or growth < 25%.

Salvage therapy was individualized according to the decision of the attending clinician.

### Statistical analysis

The primary end points were progression-free and overall survival. Secondary end points were response and toxicity.

Progression-free (PFS) and overall survival (OS) were calculated using the method of Kaplan–Meier, comparisons of survival distributions were made with the log-rank test, $\alpha = 0.05$. Since the stated aim of the study was to determine whether treatment with IFN-γ improved outcome, one-tailed *P*-values in univariate analysis are reported. PFS was defined as the interval between the initial surgery and last follow-up evaluation, diagnosis of progression or death from disease, whichever occurred first. OS was calculated according to the date of last contact alive or death from any cause. Calculation of the test power was based upon a 0.05 level one-tailed log-rank test for equality of survival curves.

Treatment group, size of residual tumour (none vs < 2 cm vs ≥ 2 cm), FIGO stage (Ic–IIc vs III–IV), histological type (serous, mucinous, endometroid vs clear cell, undifferentiated), grading (I vs II vs III), ascites at initial surgery (none vs < 500 ml vs > 500 ml) and age were included as covariates in a Cox regression analysis of PFS and OS. Comparisons of risk were based on the Wald test. Two-tailed *P*-values are reported, because the level of significance was equal for all covariates, which were tested with regard to their influence on survival either way. Differences in proportions of toxic effects and distributions of baseline characteristics and response groups were analysed by the two-tailed $\chi^2$ or Fisher's exact test when appropriate. The Wilcoxon's rank sum test was utilized to compare medians.

Data processing was performed by means of SPSS for Windows (SPSS Inc., Chicago, IL, USA).

### RESULTS

### Patients

Between December 1991 and April 1997, 148 women were enrolled in the study and randomly assigned to receive either IFN-γ with cyclophosphamide (CP) or CP alone. Randomization was terminated before the originally planned number of 200 patients was reached, because meanwhile the standard of care had changed to a platinum- and taxane-based therapy. A total of 15 patients were ineligible (ten in the group with IFN and five in the CP-only group) for the following reasons: borderline tumour (in three cases), histology other than epithelial ovarian cancer (in two), FIGO stage that did not meet the inclusion criteria (in seven), insufficient renal function (in one) and consent withdrawn and no more contact after surgery (in two). Table 1 shows the characteristics of the remaining 133 eligible patients. There were no significant differences between the study groups with respect to important prognostic parameters, as shown in Table 1. Three major protocol violations occurred among the 68 eligible patients of the CP-only group: One patient received cisplatin combined with paclitaxel instead of cyclophosphamide and in two cases cisplatin was substituted with carboplatin. In the IFN group with 65 patients carboplatin instead of cisplatin was used in two patients. According to the intent-to-treat principle all 133 eligible patients were included in the efficacy analysis.

**Table 1** Patient characteristics

| Characteristics | Treatment: CP plus IFN-γ (*n* = 65) *n* | % | CP (*n* = 68) *n* | % |
|---|---|---|---|---|
| Age (median, range) | 56, 21–75 | | 56, 22–75 | |
| FIGO stage | | | | |
| Ic | 7 | 11 | 11 | 16 |
| II | 9 | 14 | 6 | 9 |
| III | 48 | 74 | 47 | 69 |
| IV (malignant pleural effusion only) | 1 | 1 | 4 | 6 |
| Residual tumour | | | | |
| No gross residual disease after surgery | 27 | 41 | 25 | 37 |
| Small residual disease (< 2 cm) | 22 | 34 | 27 | 40 |
| Bulky residual disease (≥ 2 cm) | 16 | 25 | 16 | 23 |
| Histological type | | | | |
| Serous | 45 | 70 | 45 | 66 |
| Mucinous | 9 | 14 | 14 | 21 |
| Endometrioid | 3 | 5 | 6 | 9 |
| Clear cell | 2 | 3 | 2 | 3 |
| Undifferentiated | 5 | 8 | 1 | 1 |
| Grading | | | | |
| I | 11 | 18 | 10 | 15 |
| II | 30 | 49 | 30 | 45 |
| III | 20 | 33 | 27 | 40 |
| Unknown | 4 | | 1 | |
| Ascites | | | | |
| None | 24 | 38 | 31 | 48 |
| < 500 ml | 17 | 27 | 14 | 22 |
| > 500 ml | 22 | 35 | 19 | 30 |
| Unknown | 2 | | 4 | |

### Dose intensity (Table 2)

Of 61 eligible patients in the CP-only group, who did not discontinue therapy due to progression of disease, 64% completed six cycles as scheduled. On the other hand, 53% of 59 responding patients in the IFN group received six cycles of the combined treatment. In the IFN group only cycles with full IFN and cisplatin/cyclophosphamide treatment were counted. Although in the IFN group a lower percentage of patients completed treatment as scheduled, the difference was not statistically significant ($P = 0.75$). In addition, the dose of cisplatin was reduced in 18% of sixth CP plus IFN-γ cycles and in 10% of such cycles in the control group (n.s.).

### Response (Table 3)

Response was assessed in 81 patients with residual disease after initial surgery or disease progression under first-line therapy.

 © 2000 Cancer Research Campaign

**Table 2** Dose intensity. Percentage of eligible patients receiving cisplatin/cyclophosphamide plus IFN-γ or cisplatin/cyclophosphamide according to treatment schedule during each course and percentage of cisplatin courses with full initial dose

| Treatment course | Patients receiving assigned therapy | | Cisplatin dose | |
|---|---|---|---|---|
| | IFN group | Control | IFN group | Control |
| | % of patients | | % of cycles with initial dose | |
| 1 | 97 | 97 | 100 | 100 |
| 2 | 85 | 89 | 95 | 100 |
| 3 | 80 | 82 | 89 | 98 |
| 4 | 73 | 77 | 86 | 98 |
| 5 | 64 | 75 | 86 | 96 |
| 6 | 53 | 64 | 82 | 90 |

Clinical criteria were used as described in Patients and Methods. Of the IFN-γ treatment arm 38 patients were evaluable; 26 (68%) reached clinical complete response (CR), in contrast to 24 of 43 (56%) in the control group (Table 3). Partial response (PR) was achieved by 11% versus 21%, and disease progression was found in 21% versus 23% resulting in overall response rates of 79% and 77% (n.s.). No significant differences were found, but 50% of IFN-treated patients with bulky residual tumour achieved CR (eight of 16) compared to 19% in the control arm (three of 16). The figures for PR are 19% and 31%, respectively, resulting in overall response rates of 69% versus 50% (n.s.).

### **Toxicity** (Table 4)

As shown in Table 4a, haematological toxicity was found to be equal between treatment cycles with CP alone and those combined with IFN-γ. Also, no enhanced nephro- or hepatotoxicity was observed in the treatment arm. Severe emesis (WHO grades 3 and 4) occurred in equal proportions of treatment cycles in both groups. As demonstrated in Table 4b, neuropathy was also not more frequent in IFN-γ-treated patients.

Most patients with IFN-γ therapy suffered from a flu-like syndrome, which consisted mostly of cephalea, fatigue, chills and fever. Characteristically, symptoms set on shortly after IFN injection and lasted for several hours. There was no mitigation in the course of repeated applications.

Thrombo-embolic complications occurred with equal frequencies in the IFN-γ and control group (6% and 5%).

Additional adverse events not listed in Table 4 in the IFN treatment group and possibly associated with treatment included a newly diagnosed diabetes mellitus type II, a septicaemia caused by *Escherichia coli* and an AV block grade II–III, the latter two leading to discontinuation of IFN treatment. One patient experienced a severe depression that abated after discontinuation of IFN-γ.

**Table 3** Clinical response of 81 evaluable patients with residual disease after primary surgery or patients with disease progression under first-line therapy.

| *n* = 81 | Response[a] *n* = 38 | CP plus IFN-γ *n* = 43 | | CP only | | *P* |
|---|---|---|---|---|---|---|
| | | *n* | % | *n* | % | |
| All patients | CR | 26 | 68 | 24 | 56 | |
| evaluable for | PR | 4 | 11 | 9 | 21 | 0.38 |
| response | PD | 8 | 21 | 10 | 23 | |
| Residual tumour | | *n* = 22 | | *n* = 27 | | |
| < 2 cm | CR | 18 | 82 | 21 | 78 | |
| | PR | 1 | 4 | 4 | 15 | 0.41 |
| | PD | 3 | 14 | 2 | 7 | |
| **≥ 2 cm** | | *n* = 16 | | *n* = 16 | | |
| | CR | 8 | 50 | 3 | 19 | |
| | PR | 3 | 19 | 5 | 31 | 0.17 |
| | PD | 5 | 31 | 8 | 50 | |

[a]CR, complete response; PR, partial response; PD, progressive disease.

### Survival

Median follow-up was 29 and 24 months for overall (OS) and progression-free survival (PFS) respectively. Of 65 patients randomized to receive IFN-γ, 27 had disease progression as compared with 43 of 68 control patients. Median times to progression were 48 and 17 months, respectively, corresponding to rates of 51 versus 38% of 3-year PFS (Figure 1, $P = 0.031$, one-tailed).

The two groups did not differ significantly in OS: 18 patients who received IFN-γ died and 21 deaths occurred in the control group. Median survival has not yet been reached, neither in the IFN-γ nor in the control group and 3-year OS is 74 and 58% (Figure 2, $P = 0.23$, one-sided). However, with a sample size of 65 and 68 patients in each group, a 0.05 level one-sided log-rank test for equality of survival curves will only have a 65% power to detect a difference between the above stated proportions after 3 years observation time ($\beta = 0.35$). If the originally planned sample size of 200 had been reached (100 patients per group), the test power would have been 81%.

**Table 4a.** Frequency of toxic effects according to treatment cycles

| WHO grade | 1/2 | | 3/4 | |
|---|---|---|---|---|
| Treatment arm | + IFN-γ | CP | +IFN-γ | CP |
| | (% of treatment cycles) | | | |
| Anaemia | 21 | 24 | 0 | 0 |
| Neutropenia | 25 | 20 | 1 | 0 |
| Thrombocytopenia | 2 | 1 | 0 | 1 |
| Creatinine | 1 | 1 | 0 | 0 |
| Bilirubin | 1 | 1 | 0 | 0 |
| SGOT | 3 | 3 | 0 | 0 |
| Emesis | 76 | 73 | 14 | 14 |
| Alopecia | 48 | 48 | 10 | 14 |

**Table 4b** Frequency of toxic effects according to patients

| WHO-grade | 1/2 | | 3/4 | |
|---|---|---|---|---|
| Treatment arm | + IFN-γ | CP | +IFN-γ | CP |
| | (% of patients) | | | |
| Neurotoxicity including ototoxicity | 30 | 36 | 4 | 2 |
| Viral infection | 8 | 2 | 0 | 2 |
| Fever | 74 | 9 | 3 | 2 |
| Flu-like syndrome[a] | 66 | 2 | 3 | 0 |
| Thrombosis[b] | 6 | 5 | | |

[a]Symptoms including cephalea, fatigue, chills, myalgia. [b]WHO grading not relevant.

© 2000 Cancer Research Campaign

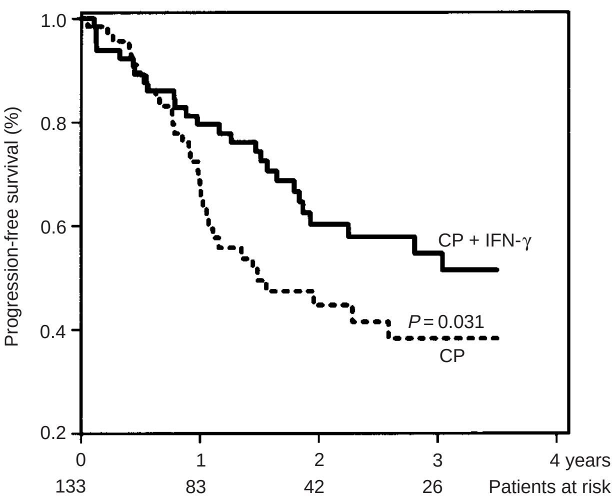

**Figure 1** Progression-free survival by treatment arm

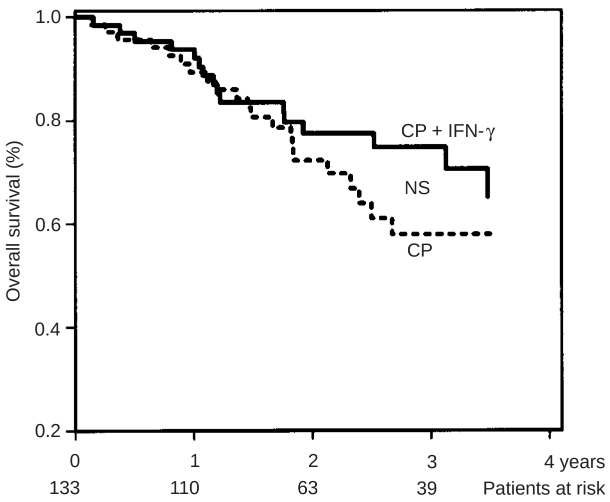

**Figure 2** Overall survival by treatment arm

**Table 5** Cox regression analysis of survival including treatment group, amount of residual tumour, age, FIGO stage, ascites, histological type and grading as covariates.

| Factors associated with survival | | RR | 95% CI[b] | P[a] |
|---|---|---|---|---|
| *Progression-free survival* | | | | |
| IFN-γ treatment | No | 1.0 | | 0.007 |
| | Yes | 0.48 | 0.28–0.82 | |
| Size of residual tumour | None | 1.0 | | 0.0000 |
| | < 2 cm | 3.5 | 1.7–7.4 | |
| | ≥ 2 cm | 7.7 | 3.7–16.0 | |
| Age | < 55 years | 1.0 | | 0.05 |
| | ≥ 55 years | 1.7 | 1.0–2.9 | |
| *Overall survival* | | | | |
| Size of residual tumour | None | 1.0 | | 0.0007 |
| | < 2 cm | 8.3 | 2.4–28.2 | |
| | ≥ 2 cm | 11.0 | 3.2–37.8 | |
| Age | < 55 years | 1.0 | | 0.001 |
| | ≥ 55 years | 2.5 | 1.2–5.1 | |

RR, relative risk; CI, confidence interval. [a]All *P*-values are two-sided.

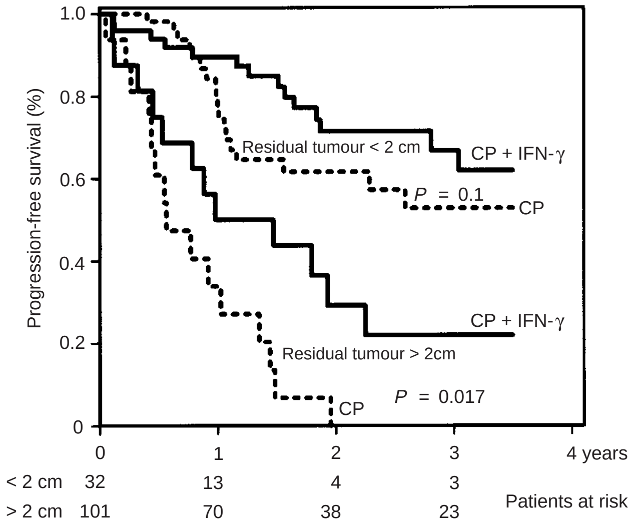

**Figure 3** Progression-free survival in patients with small residual tumour (< 2 cm) and bulky residual tumour (> 2 cm) by treatment arm

Multivariate analysis revealed that size of residual tumour and age were independent factors influencing OS and PFS when these parameters were included as covariates together with FIGO stage, ascites, histological type, grading and treatment arm. By contrast to overall survival, analysis of progression-free survival showed IFN-γ treatment to also be significantly associated with outcome with a reduction of the relative risk to 0.48 (95% confidence interval (CI) 0.28–0.82) as compared to the control group.

Table 5 shows the relative risk (RR) and *P*-values (two-tailed). To further analyse interferon-mediated effects subgroups of patients with small and large volume residual disease (< 2 cm and > 2 cm) have been evaluated (Figure 3). Three-year PFS was 53% (CP only) and 66% (CP plus IFN-γ) for patients with small volume residual disease (n.s.) and none versus 22% in the group with > 2 cm residual disease ($P = 0.01$). For OS, the corresponding figures are 65–80% and 36–56% (n.s.).

### DISCUSSION

Although IFN-γ has been demonstrated to be effective in the second-line therapy of epithelial ovarian cancer, to our knowledge no trials have so far tested this substance in the first-line treatment or in combination with chemotherapy. Our randomized prospective study included 148 patients (133 evaluable) with mostly advanced disease (75% FIGO stage III or IV). After initial debulking surgery all women were treated with the same standard chemotherapy regimen, which at the time of initiation of the study was a combination of cisplatin and cyclophosphamide, while patients allocated to the study group concomitantly received low-dose IFN-γ (three times a week in alternate weeks as described in Patients and Methods) in systemic application. The initial plan to include 200 patients had to be abandoned because the standard of care changed from chemotherapy with cisplatin and cyclophosphamide to a

 © 2000 Cancer Research Campaign platinum plus taxane combination. The power of the trial to detect a 15% difference in survival after 3 years would have been 81% (one-tailed log-rank test) for 100 patients per group and is 65% for the achieved sample size of 65 per group.

We found that IFN-γ treatment significantly improved PFS from a median of 17 months in the control group to 48 months, corresponding to rates of 38% and 51% for 3 year PFS ($P = 0.031$, one-tailed log-rank test). This result was substantiated by the fact that in multivariate analysis increased PFS was also significantly associated with IFN-γ treatment (Cox regression, $P = 0.007$, two-tailed). The RR compared with the control group (RR = 1) was reduced to 0.48 (CI 0.28–0.82). For overall survival, neither univariate nor multivariate analyses showed a significant benefit, although 3-year OS was improved from 58% to 74% (median not yet reached).

Toxicity was substantially the same in both treatment groups, except for the typical side-effects known to be associated with IFN therapy. These consisted mainly of a flu-like syndrome with fever, headache, fatigue and myalgia, and characteristically set on some hours after IFN-γ administration. The symptoms were observed in most patients receiving IFN-γ but were generally mild (Table 4a/b). Although more patients in the study group discontinued the scheduled therapy before completing six cycles, this difference was not significant.

Response was evaluated for patients with residual tumour and those who progressed under first-line therapy (Table 3). A CR was achieved in 68% treated with IFN-γ and 56% of controls. Interestingly, 50% of patients with bulky residual disease (8/16) achieved a CR versus 19% in the control group (3/16). In the subset with small residual tumour CR were observed in 82% and 78%. Possibly due to small numbers, none of these was statistically significant. In ovarian cancer IFN-γ has been tested to date solely in the second-line therapy and mostly in i.p. application in the case of peritoneal carcinomatosis. In a phase II trial with 108 patients, Pujade-Lauraine et al (1996) reported a response rate of 43% for patients with small volume residual disease (27/63). In contrast, only four of 35 (11%) patients with tumours > 2 cm responded. These results are supported by a smaller study by Colombo et al (1992) of eight patients with small-volume residual disease (< 1 cm) with three of eight patients responding. Similarly, in a study by Berek et al (1985) i.p. IFN-α showed efficacy only in small-volume i.p. disease, while no response was seen in tumours > 2 cm. This is in accordance with a general observation, that immunotherapeutic agents are more successful in patients with small tumour burden.

In contrast, in the present study, there was equal efficacy in patients with small and large tumour burden, although subset analysis is limited because of small numbers of patients. As stated above, response rates were improved also in patients with bulky disease. Improvement in survival, especially PFS, was also seen in both groups to a similar extent. The combination with chemotherapy might augment the response to IFN in accordance with the results of Weisenthal et al (1991), who found enhanced tumour-specific cytotoxic effects by IFN-γ in tumour specimens, that were obtained from patients previously treated with and responding to chemotherapy, as compared to not treated or not responding tumours. Possibly, the massive release and processing of tumour antigens primes the immune system to respond to exogenous activating signals with more potent anti-tumour effects.

Our results therefore warrant further testing of IFN-γ in the therapy of ovarian cancer. IFN-γ combined with the present standard chemotherapy of a platinum and a taxane compound particularly deserves to be evaluated.

### ACKNOWLEDGEMENTS

We thank other authors and additional institutions with their principal investigators: Elisabeth Kapshammer MD and Romeo Halbweis MD (Hospital Barmherzige Schwestern Linz), Barbara Lahodny MD (St Poelten Hospital), Susanne Groblschegg MD and Michaela Blach MD (Hospital Waidhofen an der Thaya), Dietmar Pacher MD (Villach Hospital), Lothar Schiller MD (Voecklabruck Hospital), Roland Pavelka MD (Oberwart Hospital) and Walter Neunteufel MD (Dornbirn Hospital), all Austria. We also thank Dr H Ulmer from the Institute of Biostatistics, University of Innsbruck, Austria, for assistance in statistical analyses.

### REFERENCES

Allavena P, Peccatori F, Maggioni D, Erroi A, Sironi M, Colombo N, Lissoni A, Galazka A, Meiers W, Mangioni C and Mantovani A (1990) Intraperitoneal recombinant γ-interferon in patients with recurrent ascitic ovarian carcinoma: modulation of cytotoxicity and cytokine production in tumor-associated effectors and of major histocompatibility antigen expression on tumor cells. *Cancer Res* **50**: 7318–7323

Aulitzky W, Gastl G, Aulitzky WE, Nachbaur K, Lanske B, Kemmler G, Flener R, Frick J and Huber C (1987) Interferon-γ for the treatment of metastatic renal cancer: dose-dependent stimulation and downregulation of beta-2 microglobulin and neopterin responses. *Immunobiology* **176**: 85–95

Berek JS, Hacker NF, Lichtenstein A, Jung T, Spina C, Knox RM, Brady J, Greene T, Ettinger LM, Lagasse LD, Bonnem EM, Spiegel RJ and Zighelboim J (1985) Intraperitoneal recombinant α-interferon for ‘salvage’ immunotherapy in stage III epithelial ovarian cancer: a Gynecologic Oncology Group study. *Cancer Res* **45**: 4447–4453

Billiau A (1996) Interferon-γ: biology and role in pathogenesis. *Adv Immunol* **62**: 61–130

Boehm U, Klamp T, Groot M and Howard JC (1997) Cellular responses to interferon-γ. *Annu Rev Immunol* **15**: 749–795

Colombo N, Peccatori F, Paganin C, Bini S, Brandley M, Mangioni C, Mantovani A and Allavena P (1992) Anti-tumor and immunomodulatory activity of intraperitoneal IFN-gamma in ovarian carcinoma patients with minimal residual tumor after chemotherapy. *Int J Cancer* **51**: 42–46

D’Acquisto R, Markman M, Hakes T, Rubin S, Hoskins W and Lewis JL (1988) A phase I trial of intraperitoneal recombinant gamma-interferon in advanced ovarian carcinoma. *J Clin Oncol* **6**: 689–695

Dighe AS, Richards E, Old LJ and Schreiber RD (1994) Enhanced in vivo growth and resistance to rejection of tumor cells expressing dominant negative IFN-γ receptors. *Immunity* **1**: 447–456

Hancock MC, Langton BC, Chan T, Toy P, Monahan JJ, Mischak RP and Shawver LK (1991) A monoclonal antibody against the c-erbB-2 protein enhances the cytotoxicity of cis-diaminedichloroplatinum against human breast and ovarian tumor cell lines. *Cancer Res* **51**: 4575–4580

Kaplan DH, Shankaran V, Dighe AS, Stockert E, Aguet M, Old LJ and Schreiber RD (1998) Demonstration of an interferon γ-dependent tumor surveillance system in immunocompetent mice. *Proc Natl Acad Sci USA* **95**: 7556–7561

Kleinerman ES, Kurzrock R, Wyatt D, Quesada JR, Gutterman JU and Fidler IJ (1986) Activation or suppression of the tumoricidal properties of monocytes from cancer patients following treatment with recombinant γ-interferon. *Cancer Res* **46**: 5401–5405

Malik STA, Knowles RG, East N, Lando D, Stamp G and Balkwill FR (1991) Antitumor activity of γ-interferon in ascitic and solid tumor models of human ovarian cancer. *Cancer Res* **51**: 6643–6649

Maluish AE, Urba WJ, Longo DL, Overton WR, Coggin D, Crisp ER, Williams R, Sherwin SA, Gordon K and Steis RG (1988) The determination of an immunologically active dose of interferon-gamma in patients with melanoma. *J Clin Oncol* **6**: 434–445

Mannel DN and Falk W (1983) Interferon-gamma is required in activation of macrophages for tumor cytotoxicity. *Cell Immunol* **79**: 396–402

© 2000 Cancer Research Campaign

Marth C, Fuith LC, Böck G, Daxenbichler G and Dapunt O (1989) Modulation of ovarian carcinoma tumor marker CA-125 by γ-interferon. *Cancer Res* **49**: 6538–6542

Marth C, Müller-Holzner E, Greiter E, Cronauer MV, Zeimet AG, Doppler W, Eibl B, Hynes NE and Daxenbichler G (1990) γ-interferon reduces expression of the protooncogen c-erbB-2 in human ovarian carcinoma cells. *Cancer Res* **50**: 7037–7041

Marth C, Zeimet AG, Herold M, Brumm C, Windbichler G, Müller-Holzner E, Offner F, Feichtinger H, Zwierzina H and Daxenbichler G (1996) Different effects of interferons, interleukin-1β and tumor necrosis factor-α in normal (OSE) and malignant human ovarian epithelial cells. *Int J Cancer* **67**: 826–830

Marth C, Widschwendter M, Kærn J, Jørrgensen N-P, Windbichler G, Zeimet AG, Tropé C and Daxenbichler G (1997) Cisplatin resistance is associated with reduced interferon-γ-sensitivity and increased HER-2 expression in cultured ovarian cancer cells. *Br J Cancer* **76**: 1328–1332

McGuire WP, Hoskins WJ, Brady MF, Kucera PR, Partridge EE, Look KY, Clarke-Pearson DL and Davidson M (1996) Cyclophosphamide and cisplatin compared with paclitaxel and cisplatin in patients with stage III and stage IV ovarian cancer. *N Engl J Med* **334**: 1–6

Nehmé A, Julia AM, Jozan S, Chevreau C, Bugat R and Canal P (1994) Modulation of cisplatin cytotoxicity by human recombinant interferon-gamma in human ovarian cancer cell lines. *Eur J Cancer* **30A**: 550–525

Ozols RF, Rubin SC, Thomas G and Robboy S (1997) Epithelial ovarian cancer. In: *Principles and Practice of Gynecologic Oncology*, 2nd edn, Hoskins WJ, Perez CA and Young RC (eds), pp. 919–986. Lippincott-Raven: Philadelphia

Pujade-Lauraine E, Guastalla JP, Colombo N, Devillier P, François E, Fumoleau P, Monnier A, Nooy M, Mignot L, Bugat R, Marques C, Mousseau M, Netter G, Maloisel F, Larbaoui S and Brandely M (1996) Intraperitoneal recombinant interferon gamma in ovarian cancer patients with residual disease at second look laparotomy. *J Clin Oncol* **14**: 343–350

Saito T, Berens ME and Welander CE (1986) Direct and indirect effects of human recombinant gamma-interferon on tumor cells in a clonogenic assay. *Cancer Res* **46**: 1142–1147

Weisenthal LM, Dill PL and Pearson FC (1991) Effect of prior cancer chemotherapy on human tumor-specific cytotoxicity in vitro in response to immunopotentiating biologic response modifiers. *J Natl Cancer Inst* **83**: 37–42

Welander CE, Homesley HD, Reich SD and Levin EA (1988) A phase II study of the efficacy of recombinant interferon gamma in relapsing ovarian adenocarcinoma. *Am J Clin Oncol* **11**: 465–469

*© 2000 Cancer Research Campaign*
